# Thermal tolerance and preference are both consistent with the clinal distribution of house fly proto‐Y chromosomes

**DOI:** 10.1002/evl3.248

**Published:** 2021-07-21

**Authors:** Pablo J. Delclos, Kiran Adhikari, Oluwatomi Hassan, Jessica E. Cambric, Anna G. Matuk, Rebecca I. Presley, Jessica Tran, Vyshnika Sriskantharajah, Richard P. Meisel

**Affiliations:** ^1^ Department of Biology and Biochemistry University of Houston Houston Texas 77004; ^2^ School of Biomedical Informatics University of Texas Health Science Center at Houston Houston Texas 77030

**Keywords:** genotype‐by‐environment effects, *Musca domestica*, polygenic sex determination, sex chromosomes, thermoregulation

## Abstract

Selection pressures can vary within localized areas and across massive geographical scales. Temperature is one of the best studied ecologically variable abiotic factors that can affect selection pressures across multiple spatial scales. Organisms rely on physiological (thermal tolerance) and behavioral (thermal preference) mechanisms to thermoregulate in response to environmental temperature. In addition, spatial heterogeneity in temperatures can select for local adaptation in thermal tolerance, thermal preference, or both. However, the concordance between thermal tolerance and preference across genotypes and sexes within species and across populations is greatly understudied. The house fly, *Musca domestica*, is a well‐suited system to examine how genotype and environment interact to affect thermal tolerance and preference. Across multiple continents, house fly males from higher latitudes tend to carry the male‐determining gene on the Y chromosome, whereas those from lower latitudes usually have the male determiner on the third chromosome. We tested whether these two male‐determining chromosomes differentially affect thermal tolerance and preference as predicted by their geographical distributions. We identify effects of genotype and developmental temperature on male thermal tolerance and preference that are concordant with the natural distributions of the chromosomes, suggesting that temperature variation across the species range contributes to the maintenance of the polymorphism. In contrast, female thermal preference is bimodal and largely independent of congener male genotypes. These sexually dimorphic thermal preferences suggest that temperature‐dependent mating dynamics within populations could further affect the distribution of the two chromosomes. Together, the differences in thermal tolerance and preference across sexes and male genotypes suggest that different selection pressures may affect the frequencies of the male‐determining chromosomes across different spatial scales.

**Impact Statement**

Genetic variation within species can be maintained by environmental factors that vary across the species’ range, creating clinal distributions of alleles responsible for ecologically important traits. Some of the best examples of clinal distributions come from temperature‐dependent phenotypes, such as thermal tolerance and preference. Although genotype and developmental temperature strongly affect physiological and behavioral traits in ectotherms, the correlation between these traits across genotypes and sexes within species is greatly understudied. We show that two different male‐determining chromosomes found in natural populations of house flies affect both thermal tolerance and preference in a way that is concordant with their clinal distributions across latitudes. This provides strong evidence that temperature variation across the species range contributes to the maintenance of the polymorphism. Furthermore, we find evidence that thermal preference is sexually dimorphic, suggesting that temperature‐dependent mating dynamics could further affect the distribution of genetic variation in this system. Therefore, at a macrogeographical scale, the differences in thermal tolerance and preference across male genotypes likely contribute to the maintenance of the cline. Within populations, differences in thermal preference likely affect sexual selection dynamics, which may further affect the frequencies of the chromosomes.

Ecological variation across a species’ range can select for local adaptation within populations, which can contribute to the maintenance of genetic variation by favoring different alleles across the range (Levene 1953; Felsenstein 1976; Hedrick et al. 1976; Kawecki and Ebert 2004). In addition, heterogeneous selection pressures that are distributed as a gradual continuum from one end of the species’ range to another can create a cline of genetic variation responsible for phenotypes under selection (Slatkin 1973; Endler 1977). Some of the best examples of latitudinal clines come from temperature‐dependent phenotypes (e.g., body size, developmental rate, and thermal tolerance) that have been well‐documented in flies (Partridge et al. 1994; Eanes 1999; Robinson and Partridge 2001; Hoffmann et al. 2002). Moreover, heterogeneous selection pressures across a cline may affect males and females differently (Connallon 2015; Connallon et al. 2019), although the empirical evidence for such variation in sex‐specific selection across geographic ranges is mixed (Delcourt et al. 2009; Delph et al. 2011; Allen et al. 2017; Lasne et al. 2018).

Thermal adaptation within populations and across a species range can occur via selection on physiological, anatomical, or behavioral traits. For example, north‐south gradients in heat and cold tolerance have been observed in *Drosophila* (Hoffmann et al. 2002), suggesting physiological adaptation to thermal environments. In addition, ectotherms, such as flies, rely on behavioral mechanisms of thermoregulation by avoiding suboptimal temperatures in search of more optimal ones (Dillon et al. 2009; Kearney et al. 2009), and thermal preference may be correlated with optimal thermal performance (Dawson 1975; Angilletta et al. 2002).

Concordance across genotypes between different thermal traits could reinforce the response to selection, whereas negative correlations could constrain adaptation (Etterson and Shaw 2001). However, it is not clear if physiological and behavioral thermal traits are genetically correlated within a species, between sexes, or across populations (Dawson 1975; Angilletta et al. 2002; Gilbert and Miles 2017). For example, experiments in *Drosophila subobscura* identified individual chromosomes that affected thermal tolerance or temperature preference, but no single chromosome affected both physiological and behavioral phenotypes (Dolgova et al. 2010; Rego et al. 2010; Castañeda et al. 2019). Furthermore, temperature‐dependent traits can affect assortative mating and male reproductive success (Dolgin et al. 2006; Keller and Seehausen 2012), suggesting intersexual differences in thermoregulation could affect genetic variation within populations via sexual selection. These sex‐specific selection pressures could also contribute to the maintenance of genetic variation via intersexual conflict or context‐dependent selection (Kotiaho et al. 2001; Rostant et al. 2015; Meisel 2018). Despite the importance of intersexual differences, previous work did not test for differences in the genetic correlation of thermal traits between males and females.

We used a sex chromosome polymorphism in the house fly, *Musca domestica*, to investigate the concordance of thermal tolerance and preference across clinally distributed male genotypes. House fly has a polygenic sex‐determination system, in which a male‐determining gene has been mapped to all six chromosomes, some males can carry multiple male‐determining chromosomes, and a female‐determining allele segregates on one chromosome (McDonald et al. 1978; Inoue and Hiroyoshi 1986; Dübendorfer et al. 2002; Hediger et al. 2010). The *M. domestica male determiner* (*Mdmd*) gene is most commonly found on either the third chromosome (III^M^) or what was historically referred to as the Y chromosome (Y^M^) (Hamm et al. 2014; Sharma et al. 2017). Both III^M^ and Y^M^ are very young proto‐Y chromosomes that are minimally differentiated from their homologous proto‐X chromosomes (Meisel et al. 2017; Son et al. 2019; Son and Meisel 2021). Y^M^ and III^M^ are distributed along latitudinal clines on multiple continents in the Northern Hemisphere (Tomita and Wada 1989; Hamm et al. 2005; Kozielska et al. 2008). Y^M^ is most frequently found at northern latitudes, and III^M^ is more common at southern latitudes (Fig. 1A). This distribution suggests that the Y^M^ chromosome confers higher fitness in colder climates, and, conversely, III^M^ confers higher fitness in hotter climates. Therefore, variation in temperature across the species range may create heterogeneous selection pressures that maintain the proto‐Y chromosome cline in house fly. Consistent with this hypothesis, seasonality in temperature is the best predictor of the frequencies of the proto‐Y chromosomes across natural populations (Feldmeyer et al. 2008).

**Figure 1:**
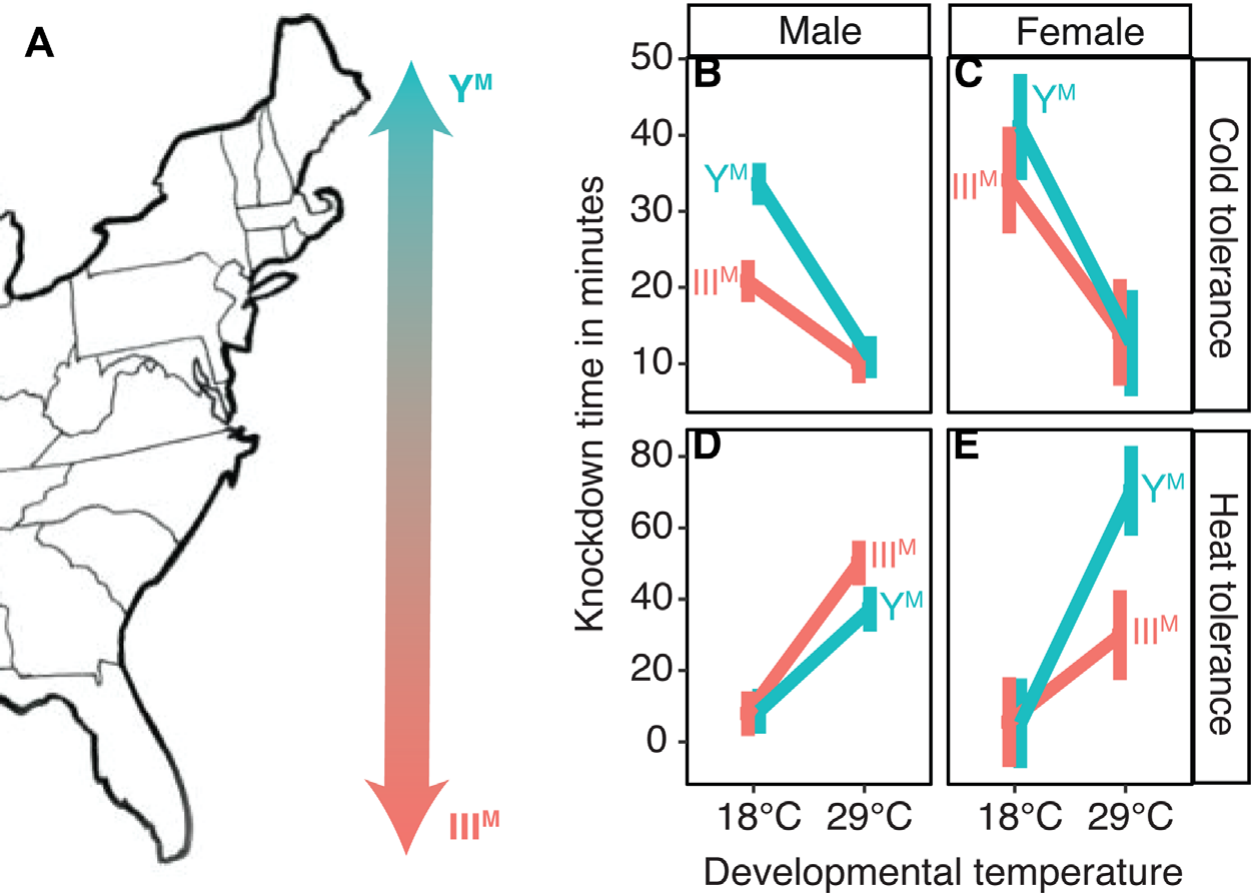
Thermal tolerance in males and females. (A) Map of the eastern United States, showing the cline of Y^M^ (more common in the north) and III^M^ (more common in the south). (B–E) Graphs show the effect of developmental temperature on knockdown time at either 4°C (cold tolerance) or 53°C (heat tolerance) for Y^M^ (turquoise) and III^M^ (salmon) male flies. Proto‐Y chromosome labels for females reflect whether males from the strain carry the Y^M^ or III^M^ chromosome. Mean knockdown time is plotted for each combination of genotype and temperature. Error bars represent standard error.

We tested the hypothesis that the Y^M^ chromosome confers cold‐adaptive phenotypes and III^M^ confers heat‐adaptive phenotypes in house fly males, which would be consistent with their latitudinal distributions (Tomita and Wada 1989; Hamm et al. 2005; Feldmeyer et al. 2008; Kozielska et al. 2008). To those ends, we first evaluated if males carrying the III^M^ chromosome (hereafter III^M^ males) have greater tolerance to extreme heat and if males carrying the Y^M^ chromosome (Y^M^ males) have greater cold tolerance. Second, we tested if III^M^ males prefer warmer temperatures than Y^M^ males, and if males and females differ in their thermal preference. We performed all experiments using flies raised at multiple developmental temperatures because thermal acclimation strongly affects temperature‐dependent phenotypes in flies and other ectotherms (Krstevska and Hoffmann 1994; Dillon et al. 2009). Together, we evaluated if thermal preference and tolerance are aligned for sex‐linked genetic variants, tested if this alignment is consistent with the geographic distribution of the proto‐Y chromosomes, and then discuss how these temperature‐dependent phenotypes could affect the access of males to female mates.

## Materials and Methods

### FLY STRAINS AND REARING

We performed our experiments using five nearly isogenic house fly strains, three with III^M^ males and two with Y^M^ males (Supporting Information Methods). All five strains have a common genetic background from an inbred III^M^ strain that was produced from a mixture of flies collected across the United States (Scott et al. 1996; Hamm et al. 2005). Each of the three III^M^ strains carries a different III^M^ chromosome from a separate wild‐derived line, and, likewise, the two Y^M^ strains carry different Y^M^ chromosomes. Each strain is fixed for its proto‐Y chromosome (either III^M^ or Y^M^), and no other sex determiners, such as the female‐determining *Md‐tra^D^* allele (Hediger et al. 2010), segregate within these strains.

We reared each strain at 18, 22, and 29°C for two generations to evaluate how thermal acclimation affects thermal tolerance (Chown and Terblanche 2006) and thermal preference (Krstevska and Hoffmann 1994; Dillon et al. 2009). Flies from each developmental temperature were assayed at equivalent physiological ages estimated by accumulated degree days (Barnard and Geden 1993; Wang et al. 2018). For our heat and cold tolerance assays, we used flies 22−50 total degree days after eclosion. For thermal preference assays, we used flies 96−115 total degree days after eclosion. Additional details and calculations are provided in the Supporting Information Methods.

### THERMAL TOLERANCE

We measured heat and cold tolerance in individual male and female house flies. To measure heat tolerance, lightly anaesthetized individual flies were transferred to a 1.5‐ml centrifuge tube that was sealed with fabric. We placed the 1.5‐ml tube in a heat block set to 53°C. This temperature was selected because it is the lowest at which heat tolerance could be measured in a reasonable period of time. The time at which a fly fell to the bottom of the tube and could not make its way back to the top was considered the knockdown time. To measure cold tolerance, lightly anaesthetized flies were transferred to a fabric‐sealed 20‐ml glass vial individually, and the vials were placed in a 4°C refrigerator with a transparent door. Knockdown occurred when a fly fell on its back to the bottom of the vial. We gently tapped the assay vial every 2−3 minutes to ensure flies were active.

For both heat and cold tolerance assays, we performed an analysis of variance (ANOVA) using the lmer() function in the lme4 (version 1.1) R package (Bates et al. 2015) to model the effect of genotype (G: Y^M^ vs. III^M^), developmental temperature (T: 18°C or 29°C), and their interaction on knockdown time (K):

K∼G+T+G×T+B+S,

with experimental batch (B) and strain (S) treated as random effects. We also constructed another model excluding the interaction term:

K∼G+T+B+S.

We then used a drop in deviance test to compare the fit of the models with and without the interaction term using the anova() function in R. We also compared heat and cold tolerance between males raised at 22 and 29°C, using the same approaches as described above. As the thermal tolerance comparisons between flies raised at 18 and 29°C and between flies raised at 22 and 29°C were conducted in separate experimental batches, we analyzed each comparison separately. Additional details are provided in the Supporting Information Methods.

### THERMAL PREFERENCE

We measured thermal preference as the position of individual flies along a 17−38°C thermal gradient (Fig. S1), following a slightly modified version of previous protocols (Anderson et al. 2013; Lynch et al. 2018). For each individual fly, we report mean thermal preference (*T_pref_*) as the average position during a 10‐minute assay window (measured once per minute). We also report thermal breadth, *T_breadth_* (Carrascal et al. 2016), as the coefficient of variation of individual‐level *T_pref_* during the assay window. *T_breadth_* provides an estimate of how individuals use thermal space within their environment (Slatyer et al. 2013). Choosier individuals show a lower *T_breadth_* value and, thus, would be expected to occupy a narrower range of temperatures within a given thermal habitat.

To determine the effects of developmental temperature (18, 22, and 29°C), genotype (Y^M^ and III^M^), and their interaction on mean *T_pref_* across sexes, we created a mixed‐effects model using the lme4 package (version 1.1) in R (Bates et al. 2015). Developmental temperature, genotype, and their interaction were included as fixed effects, and strain, batch, and lane in the thermal gradient (L) were included as random effects:

$$T_{pref}∼G+T+G\times T+B+S+L.$$

We did the same for *T_breadth_*. We then determined whether groups significantly differed in *T_pref_* or *T_breadth_* using Tukey contrasts with the multcomp package (version 1.4) in R (Hothorn et al. 2008). Within developmental temperature treatments, we used Bayesian information criterion (BIC) scores from the mclust (version 5.4.5) package in R (Scrucca et al. 2016) to determine whether the distribution of individual measures of *T_pref_* within a group is best explained by one or multiple normal distributions.

## Results

### THERMAL TOLERANCE DEPENDS ON DEVELOPMENTAL TEMPERATURE AND MALE GENOTYPE

We measured extreme heat (53°C) and cold (4°C) tolerance as a readout of differences in physiological thermal adaptation between Y^M^ and III^M^ house fly males. We observed the expected effect of acclimation on both heat and cold tolerance (Chown and Terblanche 2006): flies raised at 18°C tolerate cold longer than the flies raised at 29°C, and flies raised at 29°C tolerate heat longer than flies raised at 18°C (Fig. 1). We also find that Y^M^ males are more cold tolerant, and III^M^ males are more heat tolerant, consistent with the latitudinal distributions of Y^M^ and III^M^ males in nature (Tomita and Wada 1989; Hamm et al. 2005; Feldmeyer et al. 2008; Kozielska et al. 2008). However, the effect of genotype on thermal tolerance depends on acclimation temperature. Specifically, a linear model with an interaction between genotype (Y^M^ or III^M^) and developmental temperature fits the cold tolerance data significantly better than a model without the interaction term (*χ*²_1_ = 19.3, *P* = 1.1 × 10^−5^). This provides evidence for a *G × T* effect on cold tolerance—Y^M^ males are more cold tolerant than III^M^ males, but only if they are raised at 18°C (Fig. 1B). There is also a significant *G × T* interaction affecting heat tolerance (*χ*²_1_ = 4.71, *P* = 0.030 comparing models with and without the interaction term): III^M^ males are more heat tolerant than Y^M^ males, but only if raised at 29°C (Fig. 1D).

We next attempted to identify a threshold temperature for the genotype‐specific benefits of acclimation by comparing heat and cold tolerance of flies raised at 22 and 29°C (instead of 18 and 29°C). We did not observe a significant effect of the interaction between developmental temperature and male genotype on extreme cold tolerance (*χ*²_1_ = 0.947, *P* = 0.331 comparing models with and without an interaction term) (Fig. S2). We therefore hypothesize that there is a threshold temperature between 18 and 22°C, below which Y^M^ males experience a greater benefit of cold acclimation than III^M^ males. In contrast, there is a significant interaction between genotype and developmental temperature on heat tolerance when comparing males raised at 22 and 29°C (*χ*²_1_ = 11.02, *P* = 9.0 × 10^−4^ comparing models with and without the interaction term) (Fig. S2). Therefore, the threshold for a genotype‐specific benefit from heat acclimation lies between 22 and 29°C.

We do not expect any difference in heat or cold tolerance across females from our different strains because all females have the same genotype, regardless of the male genotype in the strain. Indeed, a model with an interaction between developmental temperature and male genotype does not fit the female cold tolerance data better than a model without the interaction term (*χ*²_1_ = 1.46, *P* = 0.23) (Fig. 1C). There is a significant effect of developmental temperature on cold tolerance in females (*χ*²_1_ = 43.5, *P* = 4.3 × 10^−11^ comparing a model with and without developmental temperature), demonstrating that females benefit from cold acclimation regardless of male genotype (Fig. 1C). Surprisingly, there is a significant interaction between male genotype and developmental temperature on heat tolerance in females (*χ*²_1_ = 10.4, *P* = 0.0013 comparing a model with and without the interaction term). In general, females raised at warmer temperatures are more heat tolerant (Fig. 1E). However, the interaction of male genotype and developmental temperature is in the opposite direction from what would be expected based on the latitudinal distribution of Y^M^ and III^M^: females from strains with Y^M^ males that are raised at 29°C are more heat tolerant than females from III^M^ strains raised at 29°C (Fig. 1E). We thus conclude that the heat and cold tolerance differences between Y^M^ and III^M^ males are specific to males and/or the proto‐Y chromosomes (i.e., not genetic background) because we do not observe the same heat or cold tolerance differences in females from those strains (who do not carry the proto‐Y chromosomes).

### THERMAL PREFERENCE DEPENDS ON DEVELOPMENTAL TEMPERATURE AND MALE GENOTYPE

**Figure 2:**
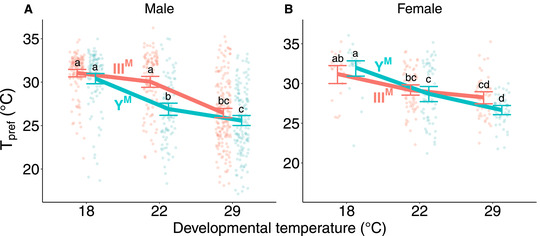
Thermal preference (*T_pref_*) of (A) male and (B) female house flies according to male genotype (III^M^ = salmon points and line, Y^M^ = turquoise points and line) and developmental temperature. Each point depicts the mean thermal preference for an individual fly, with lines and error bars denoting means within groups and standard errors of the mean, respectively. Significant differences between groups are denoted by letters, with differing letters highlighting significantly different mean thermal preferences within each graph (Tukey's post hoc test, *P* < 0.05).

We next tested if genotype and developmental temperature affect thermal preference (*T_pref_*). First, we find that *T_pref_* is inversely proportional to developmental temperature (Fig. 2), with house flies that develop at a warmer temperature preferring cooler temperatures (and vice versa), regardless of sex (male: *F*_2, 742.7_ = 138.4, *P* < 1.0 × 10^−5^; female: *F*_2, 245.3_ = 37.1, *P* = 1.19 × 10^−4^; Fig. 2). This is consistent with how developmental acclimation affects *T_pref_* in *Drosophila* (Dillon et al. 2009).

We also find that male proto‐Y chromosome genotype (Y^M^ vs. III^M^) affects *T_pref_* (*F*_1, 756.2_ = 44.5, *P* < 1.0 × 10^−5^). There is also a significant interaction effect between developmental temperature and genotype on *T_pref_* in males (*F*_2, 756.3_ = 8.47, *P* = 2.31 × 10^−4^; Fig. 2A). Male *T_pref_* is similar across genotypes when they develop at either 18 or 29°C. However, when reared at 22°C, III^M^ males prefer warmer temperatures than Y^M^ males (Tukey's post hoc test, *P* < 0.001). This is consistent with III^M^ males being more common at lower latitudes (where average temperatures are warmer) and Y^M^ males more common at higher latitudes (Tomita and Wada 1989; Hamm et al. 2005; Feldmeyer et al. 2008; Kozielska et al. 2008). We do not expect differences in *T_pref_* in females across strains because all females have the same genotype. Indeed, the genotype of males in a strain (Y^M^ vs. III^M^) and the interaction between male genotype and female developmental temperature showed no significant effect on *T_pref_* in females (ANOVA, all *P* > 0.1 in Fig. 2B). We assayed more males than females in our thermal preference experiments, and so we repeated our analysis by downsampling the data to have equal numbers of individuals across treatments. The downsampled data give equivalent results to the full dataset (Supporting Information Results).

### THERMAL BREADTH DEPENDS ON SEX AND THERMAL PREFERENCE

We used thermal breadth (*T_breadth_*) as a measure of the specificity of *T_pref_*. Male *T_breadth_* was not significantly affected by either developmental temperature, genotype, or the interaction between genotype and developmental temperature (ANOVA, all *P* > 0.1; Fig. S3A). In contrast, developmental temperature (*F*_2, 236.9_ = 16.5, *P* < 1.0 × 10^−5^), as well as the interaction between developmental temperature and male genotype (*F*_2, 243.6_ = 5.35, *P* = 0.005), had significant effects on *T_breadth_* in females. However, the significant interaction is of small effect, as females from strains with differing male genotypes do not significantly differ in *T_breadth_* within any developmental temperature treatment (Fig. S3B).

**Figure 3:**
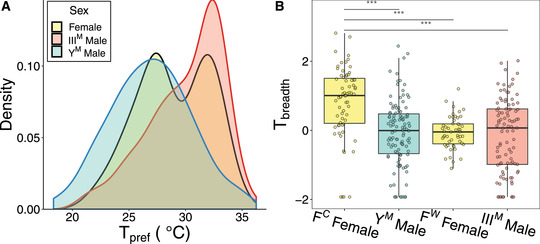
Thermal breadth (*T_breadth_*) depends on male genotype and sex. (A) Distribution of individual‐level mean thermal preferences (*T_pref_*) of III^M^ males, Y^M^ males, and pooled females that developed at 22℃. *Y*‐axis represents relative density of data points and is analogous to frequency of data points for a given *T_pref_* value. (B) *T_breadth_* of individuals raised at 22℃ according to group (F^C^ = cold‐preferring females, F^W^ = warm‐preferring females). Boxplots denote median values and lower and upper quartiles. Asterisks denote significant differences in *T_breadth_* between groups (***: Tukey's post hoc test, *P* < 0.01).

The effect of developmental temperature on *T_breadth_* in females is driven by increased variance in *T_pref_* when females develop at 22°C. The increased variance in female *T_pref_* can be explained by a mixture of two normal distributions (Fig. 3A; see Table S1 for statistics). This bimodal distribution is not a result of differences across strains because the same pattern was observed among females separately analyzed based on male genotype (Fig. S4). In comparison, a single normal distribution best fit Y^M^ male *T_pref_* when developed at 22°C, and two normal distributions best explained the III^M^ male *T_pref_* when developed at 22°C. Upon inspection, however, the two distributions representing III^M^ male *T_pref_* likely correspond to the tail (mean of 28.7°C and large variance of 10.4°C) and peak (mean of 32.6°C and small variance of 0.4°C) of a single skewed distribution, which we are unable to detect using the mclust package we used to fit distributions to our data.

We used our model‐based clustering analysis of *T_pref_* to classify individuals that developed at 22°C into one of four groups: Y^M^ males (lower *T_pref_*), III^M^ males (higher *T_pref_*), females with cooler *T_pref_* (F^C^ females, 59.3% of females tested), and females with warmer *T_pref_* (F^W^ females, 40.7% of females tested). The mean *T_pref_* of F^C^ females (26.90°C) is nearly equal to the mean *T_pref_* of Y^M^ males (26.87°C; Fig. 3A). Similarly, the mean *T_pref_* of F^W^ females (32.2°C) is near the mode of the *T_pref_* of III^M^ males (32.0−32.5°C; Fig. 3A).

We further find that *T_pref_* is predictive of *T_breadth_* for flies that develop at 22°C. We considered flies from our four *T_pref_* groups (Y^M^ males, III^M^ males, F^C^ females, and F^W^ females), and we found a significant effect of group on *T_breadth_* (*F*_3, 32.9_ = 9.40, *P* = 1.24 × 10^−4^). Specifically, F^C^ females have significantly greater *T_breadth_* than all other groups (Tukey's post hoc test, all *P* < 1.0 × 10^−5^; Fig. 3B). Therefore, if we consider *T_breadth_* as a measure of the strength of *T_pref_*, adult house flies can be summarized by one of three phenotypes related to thermal behavior when developed at 22°C: a relatively strong preference for warm temperatures (III^M^ males and F^W^ females, which have high *T_pref_* and low *T_breadth_*), a strong preference for cooler temperatures (Y^M^ males, with low *T_pref_* and low *T_breadth_*), and a relatively weak preference for cooler temperatures (F^C^ females, with low *T_pref_* and high *T_breadth_*). Downsampling the data gives similar results as the full dataset (Supporting Information Results).

## Discussion

We tested if thermal tolerance and preference depend on sex and male genotype in house flies. We find that males carrying the Y^M^ chromosome (which is common in the northern end of the species’ range) are more cold tolerant and prefer colder temperatures. Conversely, males carrying the III^M^ chromosome (which is common in the southern end of the species’ range) are more heat tolerant and prefer warmer temperatures. Our results are therefore consistent with the general trend that temperate populations are typically more cold tolerant than (sub‐) tropical ones (Gibert and Huey 2001; Hoffmann et al. 2002). The differences in thermal preference are consistent with the idea that behavioral thermoregulation can weaken selection for thermal tolerance, as predicted by the “Bogert Effect” (Huey et al. 2003; Huey and Pascual 2009; Castañeda et al. 2013). However, the fact that thermal preference and tolerance are both predicted by male genotype provides evidence that these traits are responsive to selection, suggesting any Bogert effects are not sufficient to overwhelm thermal adaptation. These differences in thermal tolerance and preference in males depend on developmental temperature, and they are not observed in congener females from the same strains (who do not carry the Y^M^ or III^M^ chromosome). However, females exhibit a bimodal *T_pref_*, with females from each of the two subgroups overlapping with one of the male genotypes.

### THERMAL TOLERANCE AND PREFERENCE DEPEND ON DEVELOPMENTAL TEMPERATURE, GENOTYPE, AND SEX

Our results demonstrate, to the best of our knowledge, the first documented example of concordant temperature preference, cold tolerance, and heat tolerance across genotypes within a species. We find that Y^M^ males both have greater cold tolerance and prefer colder temperatures, whereas III^M^ males have greater heat tolerance and prefer warmer temperatures (Figs. 1 and 2), consistent with their latitudinal distributions (Tomita and Wada 1989; Hamm et al. 2005; Feldmeyer et al. 2008; Kozielska et al. 2008). Previous work has identified concordant *T_pref_* and heat tolerance differences across species (Qu et al. 2011), or found no clear relationship between thermal tolerance and preference across genotypes within species (Yang et al. 2008; Rego et al. 2010; Castañeda et al. 2019). Body size is also predicted to vary with thermal traits (Leiva et al. 2019). In our study, we did not measure insect body size. Although we did not observe any obvious differences between strains, it is possible that some of the genotypic effect on thermal tolerance or preference we observed is due to (temperature‐dependent) morphological differences between Y^M^ and III^M^ males. Future studies should directly test this hypothesis.

We observed strong effects of developmental temperature on both thermal tolerance and preference that depend on both genotype and sex. Acclimation effects on heat and cold tolerance (Fig. 1) are well‐documented for ectotherms, including flies and other insects (Bowler and Terblanche 2008). An inverse relationship between developmental temperature and thermal preference has also been observed in other flies (Dillon et al. 2009; Castañeda et al. 2013). Behaviorally navigating toward compensatory temperatures could serve as a means of mitigating the costs of thermally suboptimal development (i.e., too hot or too cold). The observed relationships between thermal tolerance and developmental temperatures are likely to be caused by acclimation and unlikely to be the result of natural selection within our experiment for two reasons. First, there is unlikely to be sufficient genetic variation in these inbred strains for selection to generate these results within two generations. Second, prior attempts at selecting for thermal tolerance in house flies resulted in negligible differences in tolerance across developmental temperatures (Geden et al. 2019). However, it is worth noting that the males used by Geden et al. (2019) were likely all III^M^ based on their geographic origin. Had the experimental population consisted of both III^M^ and Y^M^ males, a response to tolerance may have been detected. We conclude that the differences in thermal tolerance (and preference) between Y^M^ and III^M^ males have evolved across the natural populations from which we sampled the Y^M^ and III^M^ chromosomes.

There are important methodological implications for our observation that variation in thermal preference across genotypes depends on developmental temperature. We only observe warmer (colder) thermal preferences in III^M^ (Y^M^) males when developed at 22°C; thermal preference did not differ between male genotypes when raised at more extreme (18 and 29°C) temperatures (Fig. 2A). Previous studies attempting to estimate genetic variance in thermal preference within or among populations of *Drosophila* have had mixed results. Although some studies identified genetic variance among populations within species (Good 1993; Castañeda et al. 2013), others did not detect substantial variance within (Krstevska and Hoffmann 1994) or among species (MacLean et al. 2019). Our results show that the phenotypic presentation of genetic variation for thermal preference can depend on the environmental conditions experienced, which could explain why this variance was not detected in other experiments. In addition, although the genetic mechanisms that regulate thermal tolerance in other systems have been extensively studied (Svetec et al. 2011; Königer and Grath 2018; Königer et al. 2019), it is possible that some of the molecular pathways involved will only be revealed through experiments conducted across developmental temperatures.

We identify multiple differences between males and females in their thermal tolerance and preferences. The strain differences we observed are primarily limited to males, which is expected because the males differ in genotypes (Y^M^ and III^M^) but females are isogenic (Meisel et al. 2015). However, there is a difference in heat tolerance between females from strains with Y^M^ males and females from strains with III^M^ males (Fig. 1). Although we can rule out certain genotypic explanations for this difference (i.e., all females are isogenic and do not carry *Md‐tra^D^*), we do not yet have a mechanistic explanation on why females show the opposite developmental heat tolerance from males. Nevertheless, the difference in heat tolerance observed between females from different strains is in the opposite direction as between Y^M^ and III^M^ males from those strains. This helps us to conclude that differences between Y^M^ and III^M^ males are indeed a result of different proto‐Y chromosomes rather than their genetic backgrounds.

We identified a female‐specific plasticity for thermal preference that does not map to male genotype. In females, we found that neither thermal tolerance nor thermal preference differs predictably between strains where males carry different proto‐Y chromosomes (Figs. 1C, 1E, and 2B). However, there is a bimodal thermal preference for females that develop at 22℃ (Fig. 3A), regardless of congener male genotype. In addition, females that had colder *T_pref_* when developed at 22°C also had a larger *T_breadth_* (Fig. 3B). In small ectotherms with little thermal inertia, measures of movement along a thermal gradient (such as *T_breadth_*) are predicted to be positively correlated with environmental temperature (Anderson et al. 2007). However, we observe the opposite relationship between mean environmental temperature (*T_pref_*) and *T_breadth_* in females (Fig. 3), suggesting that the difference in *T_breadth_* cannot be explained by thermal inertia. Our results suggest that, in nature, females with colder temperature preferences may occupy a wider range of temperatures than females with warmer temperature preferences. Because all females in our experiment are expected to have the same genotype, we hypothesize that these differences in *T_pref_* and *T_breadth_* are conferred by a plastic response to some yet to be characterized factor (e.g., microclimates within larval rearing containers). Alternatively, this plasticity could have a stochastic origin that is intrinsic to the development of thermal preference (Honegger and de Bivort 2018; Jensen 2018).

The correlation between thermal preference and thermal breadth at 22°C is female specific: Y^M^ and III^M^ males have similar *T_breadth_* values when raised at 22°C despite their differences in *T_pref_*. Although general sex differences in thermal tolerance (Hoffmann et al. 2005) and thermal preference (Krstevska and Hoffmann 1994) have been documented, this is the first study, to our knowledge, to identify sex differences in the relationship between thermal preference and thermal breadth. Our results suggest that male and female house flies exhibit different thermoregulatory behavioral patterns that may further be influenced by genotype. Directly identifying a sex‐by‐genotype‐by‐environment interaction is beyond the scope of this study because sex and genotype are confounded in our experimental design (the females in our experiment have a different genotype from either male, characterized by a lack of either the III^M^ or Y^M^ chromosome). Nonetheless, the house fly is a tractable system for directly testing for sex‐specific genotype‐by‐environment interactions on thermoregulation. For example, future work could test for sex‐specific effects of Y^M^ and III^M^ by measuring phenotypes in females carrying a proto‐Y chromosome along with the epistatic female‐determining *Md‐tra^D^* allele (Hediger et al. 2010; Hamm et al. 2014).

### ENVIRONMENTAL HETEROGENEITY AND THE MAINTENANCE OF POLYGENIC SEX DETERMINATION

Sex‐determination pathways rapidly diverge across species, driving evolutionary turnover of sex chromosomes (Bull 1983; Beukeboom and Perrin 2014). Polygenic sex‐determination systems, in which more than one master sex determining locus segregate independently on different chromosomes, have been observed in multiple animal species (Moore and Roberts 2013). Most population genetic models that attempt to explain the stable maintenance of polygenic sex determination focus on sexually antagonistic effects of sex‐determining loci or linked alleles on sex chromosomes (Rice 1986; van Doorn and Kirkpatrick 2007; Kozielska et al. 2010; Meisel et al. 2016). Less attention has been given to ecological factors that can maintain polygenic sex determination (Pen et al. 2010; Bateman and Anholt 2017).

Our results demonstrate how spatially variable ecological factors can maintain polygenic sex determination. Specifically, thermal tolerance and preference phenotypes conferred by the Y^M^ and III^M^ chromosomes (Figs. 1 and 2) are consistent with the clinal and temperature‐dependent distributions of the Y^M^ and III^M^ chromosomes (Tomita and Wada 1989; Hamm et al. 2005; Feldmeyer et al. 2008; Kozielska et al. 2008). Previous experiments identified multiple fitness advantages conferred by the III^M^ chromosome over Y^M^ at warmer temperatures, including an increase in frequency of III^M^ over generations in a laboratory population (Hamm et al. 2009). However, these fitness differences can only explain the invasion or fixation of the III^M^ chromosome, not the maintenance of the polymorphism. In contrast, differences in thermal tolerance and preference could maintain proto‐Y chromosome polymorphism across the species’ range, similar to how selection maintains other clinal variation (Slatkin 1973; Endler 1977).

The house fly system reveals how temperature variation can contribute to the maintenance of polygenic sex determination independently of selection on the sex‐determination pathway itself. Temperature is an important contributor to the evolution of sex‐determination pathways in vertebrates (Bull and Vogt 1979; Holleley et al. 2015). However, the effects of the house fly proto‐Y chromosomes on thermal tolerance and preference likely act independently of the sex‐determination pathway because there are not differences in the expression of sex‐determination genes across house fly male genotypes raised at different temperatures in a way that is consistent with their clinal distribution (Adhikari et al. 2020). This suggests that the effects of the Y^M^ and III^M^ chromosomes on thermal phenotypes are a result of alleles on proto‐Y chromosomes that are genetically linked to the male‐determining locus, as opposed to the male‐determiner itself. Therefore, our results highlight how temperature can be important for the evolution of sex determination independently of temperature‐dependent activity of the sex‐determination pathway. Future theoretical work should consider the effect of spatially heterogeneous selection pressures on the maintenance of polygenic sex determination, similar to how temporal heterogeneity can create fluctuating selection pressures that maintain polygenic sex determination (Bateman and Anholt 2017).

### SELECTION ON THERMAL PHENOTYPES MAY DEPEND ON GEOGRAPHICAL SCALE

**Figure 4:**
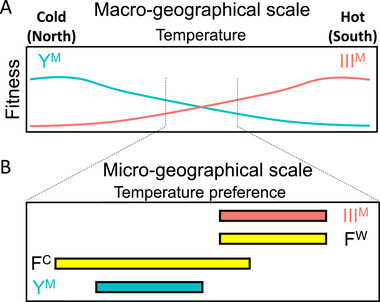
Selection on the III^M^ and Y^M^ chromosomes likely differs across geographic scales. (A) At the macrogeographical scale, selection for thermal tolerance and/or thermal preference results in the clinal distribution of the Y^M^ (turquoise) and III^M^ (salmon) chromosomes. (B) At intermediate developmental temperatures, male genotypes (Y^M^ vs. III^M^) differ in thermal preference, which may create asymmetrical mating opportunities because of variation in female thermal preference and breadth (F^C^ vs. F^W^). The asymmetry of the overlap of males and females at the intermediate developmental temperature could affect sexual selection in populations where Y^M^ and III^M^ both segregate.

Our results suggest that selection on thermal traits differs between macrogeographic species ranges and at a microgeographical scale within populations. Similar differences in selection pressures according to geographic scale have been documented before in other species (Richter‐Boix et al. 2010; De Block et al. 2013; Tüzün et al. 2017). Thermal tolerance and preference in male house flies depend on proto‐Y chromosome genotype in a way that is consistent with the latitudinal distribution of the Y^M^ and III^M^ chromosomes (Figs. 1 and 2). This suggests that, at the macrogeographic scale, selection is operating on male physiology and behavior to create or maintain the clinal distribution of Y^M^ and III^M^ (Fig. 4A). It is also worth noting that our study focuses on only two male genotypes (III^M^ and Y^M^). Although these are the most prevalent genotypes in the eastern United States, other genotypes exist (including males with multiple proto‐Y chromosomes, and females with proto‐Y and proto‐W chromosomes) and are common in other populations (Franco et al. 1982; Feldmeyer et al. 2008; Hamm and Scott 2009; Hamm et al. 2014). Future studies should characterize thermal tolerance and preference of these other genotypes to determine whether their geographical distribution is similarly explained by thermal biology.

At an intermediate developmental temperature (22°C), female thermal preference is bimodal for a reason that we have yet to determine (Fig. 3A). This raises the possibility that within populations near the center of the cline (i.e., at a microgeographic scale), where Y^M^ and III^M^ both segregate (e.g., Hamm and Scott 2008; Meisel et al. 2016), sexual selection may favor males that can preferentially obtain access to the two different female phenotypes. Although differences in thermal preference probably did not evolve in response to sexual selection, these differences do likely have important consequences on the reproductive success of III^M^ and Y^M^ males where they co‐occur. III^M^ males may disproportionately benefit from differences in *T_pref_* and *T_breadth_* between males and females. F^C^ females that prefer colder temperatures have greater *T_breadth_* than warm‐preferring F^W^ females and both male genotypes (Fig. 3B), suggesting that F^C^ females occupy a wider range of thermal habitats. Thus, III^M^ males may gain an advantage by having greater access to F^W^ females, as well as occasional access to F^C^ females, in contrast to Y^M^ males who would only be likely to encounter F^C^ females (Fig. 4B). This raises the possibility that differences in thermal preference across genotypes and sexes could affect the dynamics of sexual selection.

## AUTHOR CONTRIBUTIONS

PD, KA, and RM conceived and designed the study. KA, OH, VS, and JC collected, and KA analyzed, all thermal tolerance data. PD, RP, JT, and AM collected, and PD analyzed, all thermal preference data. PD, KA, and RM wrote the manuscript, and all authors reviewed the manuscript prior to submission.

## DATA ARCHIVING

All data files used for analyses described in this manuscript have been deposited in Dryad (https://doi.org/10.5061/dryad.n2z34tmvs). Raw video and image files from thermal preference assays are available from the authors upon request.

## Supporting information

**Figure S1**. Thermal gradient design.
**Figure S2**. Cold tolerance (**A**) and heat tolerance (**B**) in males raised at 22°C and 29°C.
**Figure S3**. Thermal breadth of (**A**) male and (**B**) female house flies according to genotype (III^M^ = salmon points and line, Y^M^ = turquoise points and line) and developmental temperature.
**Figure S4**. Distributions of individual‐level mean thermal preferences of females according to male genotype in the strain (females from strains with III^M^ males are shown in red, females from strains with Y^M^ males are shown in blue).
**Figure S5**. Summary of validation experiments for thermal preference assays.
**Table S1**. Summary statistics showing properties of mixture models fit to Tpref values for III^M^ male, Y^M^ male, and female house flies. The BIC values and summary statistics of the best fit models are shown in bold. df = degrees of freedom.
